# Revolutionizing Sleep Medicine: The Impact of Machine Learning on Diagnosis, Treatment, and Personalized Care

**DOI:** 10.1002/hsr2.72323

**Published:** 2026-05-25

**Authors:** Alexander A. Huang, Samuel Y. Huang

**Affiliations:** ^1^ Cornell University Ithaca New York USA; ^2^ School of Medicine Northwestern University Feinberg Chicago Illinois USA; ^3^ Icahn School of Medicine at Mount Sinai South Nassau New York City New York USA

## Abstract

**Background:**

Machine learning (ML) has emerged as a transformative tool in sleep medicine, offering innovative solutions for diagnosing, treating, and understanding sleep disorders. This review examines the current state and future potential of ML applications in this domain.

**Methods:**

We conducted a comprehensive review of the literature, focusing on key advancements in ML technologies applied to sleep medicine. Specific areas explored include the classification of sleep stages, treatment optimization for sleep apnea and insomnia, and the integration of wearable technology for monitoring sleep patterns.

**Results:**

ML has significantly improved the accuracy and efficiency of sleep stage classification and enabled the development of tailored treatments for sleep disorders. Wearable technology has facilitated real‐time, non‐invasive sleep monitoring. Despite these advancements, challenges such as data privacy concerns, ethical considerations, and the need for interdisciplinary collaboration remain substantial barriers to broader adoption.

**Conclusion:**

Machine learning has the potential to revolutionize sleep medicine by personalizing care and enhancing predictive models for managing sleep disorders. Overcoming existing challenges is crucial to fully realize the benefits of ML, enabling more effective and personalized approaches to diagnosis and treatment. This review highlights the transformative role of ML in sleep medicine and its capacity to improve sleep health outcomes.

## 1 Introduction

In recent years, the intersection of machine learning (ML) and healthcare has opened new avenues for diagnosing, treating, and understanding a myriad of health conditions, with sleep disorders being a prime area of focus [1, 2, 3, 4]. The importance of sleep for overall health cannot be overstated, affecting everything from cognitive function and emotional regulation to physical health and disease resistance [5, 6, 7]. Yet sleep disorders, ranging from insomnia and sleep apnea to circadian rhythm disruptions and narcolepsy, afflict a significant portion of the global population, leading to substantial health burdens and diminished quality of life [4, 8, 9].

This review paper critically evaluates the burgeoning body of research on the application of machine learning techniques to the field of sleep medicine [5, 7, 10, 11, 12]. Given the complexity and heterogeneity of sleep disorders, traditional diagnostic and treatment modalities often fall short, either due to the invasive nature of the assessment tools, the subjective nature of self‐reported symptoms, or the inability to capture the multifaceted nature of sleep in a laboratory setting [6, 13]. Machine learning, with its capacity to unearth patterns and insights from large datasets, offers a promising alternative with the potent to revolutionize the way sleep disorders are understood, diagnosed, and managed [8, 14, 15, 16]. Machine learning offers powerful opportunities to research temperamental characteristics and sleep macrostructure and microstructure enabling analysis of the auatonomic nervous system activity and amygdala‐related affective regulation which may yield clinically meaningful insights to pathways linking sleep with cardiovascular and psychiatric disorders.

The paper is structured to first provide an overview of common sleep disorders, highlighting the challenges and limitations inherent in current diagnostic and treatment approaches. It then delves into the principles of machine learning, with a focus on those techniques most pertinent to sleep research, such as supervised and unsupervised learning, deep learning, and reinforcement learning [10, 15, 17, 18, 19]. The core of the review presents a comprehensive analysis of recent studies that have applied ML to various aspects of sleep medicine, including the detection of sleep stages from polysomnography data, the identification of sleep apnea using wearable devices, the prediction of treatment outcomes for insomnia, and the exploration of genetic factors influencing sleep disorders [11, 12, 19].

Furthermore, the review critically examines the ethical considerations, data privacy concerns, and potential biases associated with the use of machine learning in healthcare, specifically in the context of sleep disorders [17, 18, 19]. The study discusses the challenges of integrating ML‐based tools into clinical practice, including the need for robust validation studies, regulatory hurdles, and the importance of clinician and patient education [15, 20, 21, 22].

In conclusion, the review underscores the transformative potential of machine learning in advancing our understanding and management of sleep disorders [23, 24, 25, 26]. It calls for a multidisciplinary approach, bringing together expertise from computer science, neuroscience, psychology, and clinical medicine, to harness the power of ML in a way that is ethical, patient‐centered, and conducive to enhancing sleep health on a global scale [27, 28, 29, 30, 31].

## 2 Methods

### 2.1 Literature Search Strategy

A comprehensive literature search was conducted using databases including PubMed, Scopus, IEEE Xplore, and Google Scholar to identify studies published up to April 2023. The search strategy employed a combination of keywords and MeSH terms related to “machine learning,” “sleep disorders,” “sleep medicine,” “predictive models,” and “wearable technology.” Filters were applied to select articles in English, with no restriction on the study design to encompass a broad spectrum of research including original research articles, review papers, and case studies.

### 2.2 Selection Criteria

Studies were included based on the following criteria: (1) application of machine learning techniques in the diagnosis, treatment, or understanding of sleep disorders; (2) studies that reported on the development or validation of ML models; and (3) research involving the use of wearable technology for sleep monitoring. Exclusion criteria were articles not in English, studies not involving human subjects, and papers focusing solely on theoretical aspects of machine learning without application to sleep medicine.

### 2.3 Data Extraction

Relevant data were extracted from the selected studies, including the type of sleep disorder, machine learning methods employed, key findings, sample size, and any noted limitations or ethical considerations. This information was categorized based on the application area within sleep medicine: diagnosis, treatment optimization, monitoring, and predictive modeling.

### 2.4 Quality Assessment

The quality of included studies was assessed using a modified version of the Newcastle Ottawa Scale (NOS) for evaluating the quality of non‐randomized studies in meta‐analyses. This assessment focused on the selection of study groups, comparability of groups, and the ascertainment of either the exposure or outcome of interest for case‐control or cohort studies respectively.

### 2.5 Data Synthesis

A narrative synthesis approach was used to summarize findings from the included studies. The synthesis focused on the impact of machine learning applications in sleep medicine, categorized by diagnostic tools, treatment optimization, monitoring applications, and predictive modeling. Challenges, such as data privacy and ethical considerations, were also synthesized to provide a comprehensive overview of the field's current state and future directions.

### 2.6 Ethical Considerations

This review did not involve primary data collection from human participants; therefore, ethical approval was not required. All analyses were based on publicly available data from previously published studies.

Through this methodological approach, this review paper aims to offer a systematic and comprehensive overview of the applications of machine learning in the field of sleep medicine, highlighting advancements, challenges, and future directions for re‐search and clinical practice.

## 3 Results

### 3.1 Overview of Selected Studies

The literature search yielded a total of 1,457 records, from which 312 studies met the inclusion criteria after title, abstract screening, and full‐text review. These studies spanned a range of sleep disorders, including insomnia, obstructive sleep apnea (OSA), narcolepsy, circadian rhythm sleep disorders, and sleep‐related movement disorders. The methodologies employed across these studies varied significantly, with a prominent use of deep learning techniques, notably Convolutional Neural Networks (CNNs) and Recurrent Neural Networks (RNNs), alongside traditional machine learning algorithms such as Support Vector Machines (SVMs) and Random Forests.

### 3.2 Machine Learning in Diagnosis and Classification

A substantial portion of the selected studies (*n* = 142) focused on the use of ML for the diagnosis and classification of sleep stages. Deep learning models, particularly CNNs, demonstrated high accuracy in classifying sleep stages from polysomnography (PSG) data, with several studies achieving over 90% accuracy. Performance was most commonly evaluated using metrics such as accuracy, sensitivity, specificity, area under the receiver operating characteristic curve (AUROC), and F1‐score. While these findings underscore the potential of ML to enhance the efficiency and accuracy of sleep disorder diagnosis, it is important to note that many high‐performing models were validated primarily using internal validation strategies, with comparatively fewer studies undergoing robust external validation. These advancements suggest the potential for ML to enhance the efficiency and accuracy of sleep disorder diagnoses.

### 3.3 Treatment Optimization and Predictive Modeling

Another critical area of application was in treatment optimization for sleep disorders (*n* = 85). ML models were effectively used to predict individual responses to treatments such as CPAP therapy for OSA and cognitive behavioral therapy for insomnia, facilitating personalized treatment approaches. Furthermore, predictive models were developed to forecast the onset of sleep disorders based on genetic, behavioral, and environmental factors, offering opportunities for early intervention.

### 3.4 Wearable Technology and Monitoring

The integration of ML with wearable technology for sleep monitoring was highlighted in 65 studies. These studies demonstrated the feasibility of using wearable devices, coupled with ML algorithms, to monitor sleep quality, detect sleep apnea episodes, and even predict sleep stages with considerable accuracy. This application represents a shift towards more accessible and non‐invasive methods for sleep disorder monitoring and management.

### 3.5 Challenges and Limitations

Despite the promising results, the studies reviewed also highlighted several challenges, including the need for large, diverse datasets to train and validate ML models, concerns over data privacy and security, and the necessity for interdisciplinary collaboration to translate ML findings into clinical practice effectively. Additionally, the variability in study methodologies and the lack of standardized reporting were identified as limitations impacting the comparability and generalizability of results. For certain populations such as those with stroke, neuropathy, and chronic pain these models can help identify complex interactions between comorbid conditions and sleep architecture especially if they are integrated with multimodal clinical data such as polysomnography, wearable devices, and electronic health record variables.

Despite the advances enabled by machine learning, ML‐based tools should be viewed as adjunctive decision support systems rather than replacements for comprehensive clinical evaluation. Automated algorithms interpreting data remain vulnerable to errors that include false negatives, misclassification, contextual blind spots and other patient behaviors that may not be recorded by the dataset. Certain situational dependent algorithmic inputs such as new stressors, medication use, or other concealed factors that are not disclosed by the individual or encoded in the data may lead to algorithmic failure. This collaborative model underscores the continued primacy of clinical judgment while leveraging ML to enhance efficiency, pattern recognition, and personalized care in sleep medicine.

### 3.6 Ethical Considerations and Future Directions

Ethical considerations in the use of ML in sleep medicine were addressed in a minority of studies, emphasizing the importance of transparency, accountability, and patient privacy in the development and deployment of ML models. The discussions on future directions pointed towards the integration of multimodal data sources, refinement of algorithms for better personalization, and the exploration of ML's potential to uncover novel insights into the pathophysiology of sleep disorders.

In conclusion, the results of this review underscore the significant potential of machine learning to transform the field of sleep medicine through enhanced diagnostic accuracy, personalized treatment plans, and innovative monitoring solutions. However, realizing this potential will require overcoming existing challenges, including ethical concerns, data limitations, and the need for standardization across studies.

## 4 Discussion

This review synthesizes findings from the final selected papers, illustrating the breadth of machine learning (ML) applications in sleep medicine across various domains. Studies have predominantly focused on diagnosing and classifying sleep dis‐orders, notably through the use of deep learning techniques such as Convolutional Neural Networks (CNNs) and Recurrent Neural Networks (RNNs), which have shown high efficacy in classifying sleep stages from polysomnography data. These advances highlight ML's potential to significantly enhance diagnostic accuracy and efficiency. Treatment optimization emerges as another key application area, with ML models being developed to predict individual responses to treatments like Continuous Positive Airway Pressure therapy for obstructive sleep apnea and cognitive behavioral therapy for insomnia, enabling more personalized treatment strategies. Furthermore, predictive modeling has been applied to anticipate the onset of sleep disorders based on a range of factors, including genetic, behavioral, and environmental influences, thereby opening avenues for early intervention.

The integration of ML with wearable technology for sleep monitoring represents a shift towards more accessible, non‐invasive methods of managing sleep disorders. Studies in this area have demonstrated the feasibility of using wearable devices, combined with ML algorithms, to accurately monitor sleep quality and detect sleep apnea episodes. Despite these promising developments, the review also highlights several challenges, such as the need for larger and more diverse datasets to train ML models, concerns over data privacy and security, and the necessity for interdisciplinary efforts to translate ML advancements into clinical practice. Additionally, ethical considerations related to the use of ML in sleep medicine were addressed, underscoring the importance of maintaining transparency, accountability, and respect for patient privacy. The variability in methodologies across studies and the lack of standardized re‐porting criteria were identified as limitations affecting the comparability and generalizability of findings. As the field moves forward, there is a clear need for refining algorithms to enhance personalization and exploring ML's potential to uncover new insights into the pathophysiology of sleep disorders, suggesting a promising yet challenging roadmap for the integration of ML into sleep medicine [23, 30, 32, 33, 34, 35]. These models excel in analyzing polysomnography (PSG) data to classify sleep stages with remarkable accuracy, using advanced algorithms like Convolutional Neural Networks (CNNs) and Recurrent Neural Networks (RNNs). These techniques are adept at identifying anomalies in sleep patterns that signal disorders such as sleep apnea, insomnia, and narcolepsy, show‐casing the potential of ML in revolutionizing diagnostic processes [36, 37, 38].

Parallel to these developments, the advent of wearable technology has significantly expanded the capability to gather sleep‐related data outside traditional clinical settings. This surge in data availability has been matched by the application of ML algorithms to scrutinize information from wearable devices, enabling the monitoring of sleep quality, detection of sleep apnea, and even the prediction of various sleep disorders. This application of ML not only enhances the ability to understand sleep patterns in real‐time but also democratizes health monitoring, making it more accessible to individuals.

Furthermore, ML models have been instrumental in predicting the outcomes of treatments for sleep disorders, such as Continuous Positive Airway Pressure (CPAP) therapy for sleep apnea and cognitive‐behavioral therapy for insomnia [39, 40]. By predicting individual responses to these treatments, ML models facilitate the creation of personalized treatment plans, thereby improving both the efficacy of interventions and patient adherence [41, 42, 43].

The scope of ML in sleep medicine also extends to the analysis of large genomic datasets, which has enabled the identification of genetic markers associated with sleep disorders and the assessment of environmental factors impacting sleep quality and disorders [24, 29, 30, 31, 32]. This genetic and environmental analysis through ML techniques provides a deeper understanding of the multifaceted nature of sleep disorders, paving the way for targeted treatment strategies [24, 25, 26, 44].

Despite these promising advancements, the application of ML in sleep medicine is not without challenges. Issues surrounding data privacy, ethical considerations in algorithmic decision‐making, and the integration of ML tools into clinical practice pose significant hurdles [36, 40, 45, 46, 47, 48]. The importance of ensuring the fairness and transparency of ML models is paramount, as these factors are critical to the acceptance and effectiveness of ML technologies in healthcare [49, 50, 51]. Addressing these challenges is essential for the continued advancement and integration of ML applications in the field of sleep medicine, highlighting the need for a balanced approach that prioritizes ethical standards, patient privacy, and the reliability of ML models [52, 53, 54, 55].

Machine learning (ML) algorithms have ushered in a new era in the assessment of sleep quality, employing automated analyses that leverage data from both polysomnography (PSG) and wearable devices [2, 56, 57, 58, 59, 60, 61]. These algorithms efficiently evaluate sleep efficiency, duration, and disturbances, providing detailed insights into sleep patterns without the traditional need for manual scoring [3, 4]. Further extending ML's utility, models have been adept at identifying disruptions in circadian rhythms by analyzing activity patterns, light exposure, and sleep timings [61, 62, 63]. This capability facilitates the diagnosis of circadian rhythm disorders, such as Delayed Sleep Phase Syndrome (DSPS) and Non‐24‐h Sleep‐Wake Disorder (N24SWD), enhancing the understanding of these conditions [45, 64, 65, 66].

In the realm of personalized medicine, ML algorithms utilize data on individuals' sleep habits, lifestyle factors, and environmental conditions to offer customized recommendations aimed at improving sleep hygiene and optimizing sleep environments [67, 68, 69]. Additionally, predictive modeling has been employed to assess the risk of developing insomnia, analyzing genetic, psychological, and environmental factors to enable early intervention strategies [49, 70, 71, 72]. Innovations in ML have also been applied to the analysis of speech patterns in individuals with sleep‐related speech disorders, such as REM Sleep Behavior Disorder (RBD), aiding in early detection and differentiation from other sleep disorders [52, 73, 74, 75].

Deep learning models have played a pivotal role in enhancing the understanding of the pathophysiology of sleep apnea by analyzing comprehensive data from PSG [54, 66, 76]. This analysis includes respiratory, cardiac, and brain activity, potentially paving the way for novel treatment approaches [68, 77, 78, 79]. ML models also contribute to the optimization of treatment plans for sleep disorders, including the fine‐tuning of CPAP machine settings for sleep apnea patients and determining the optimal timing and dosage of medications for those with insomnia or narcolepsy [41, 75, 80].

Significantly, ML algorithms have been used to monitor and predict sleep‐related breathing disorders in children, addressing conditions like obstructive sleep apnea, which often remain undiagnosed [36, 37, 38]. Moreover, ML techniques have explored the impact of mental health on sleep disorders, shedding light on the comorbidity be‐tween conditions such as anxiety, depression, and sleep disturbances, and suggesting comprehensive treatment approaches [39, 40, 81]. Lastly, there's been a focus on developing non‐invasive diagnostic tools powered by ML. These tools utilize signals from wearable devices or smartphone applications, offering a more accessible and patient‐friendly approach for the diagnosis and monitoring of sleep disorders, illustrating ML's broad and transformative impact on sleep medicine [36, 38, 43, 82, 83, 84].

Machine learning (ML) is increasingly being recognized for its potential to revolutionize sleep medicine, offering innovative solutions across a spectrum of sleep‐related challenges. In patients with neurological disorders like multiple sclerosis and Parkinson's disease, ML models are being used to analyze sleep patterns, providing valuable insights into disease progression and its impact on sleep quality [37, 40]. Adaptive algorithms have been developed to optimize treatment for obstructive sleep apnea, dynamically adjusting CPAP machine settings in real‐time to enhance treatment efficacy. In critical care settings, ML models analyze ICU monitor data to identify sleep disruptions, informing potential interventions to improve patient outcomes [41, 48, 80]. Furthermore, predictive ML algorithms are streamlining the identification of patients likely to benefit from oral appliance therapy in sleep apnea cases, reducing reliance on trial and error.

ML's applications extend to diagnosing and monitoring, where algorithms process behavioral and physiological data from wearable devices to detect daytime sleepiness and automate the detection of parasomnias like sleepwalking [39, 40, 42, 43, 75]. Genetic analysis through ML is unraveling the genetic underpinnings of sleep disorders, opening the door to genomic‐based treatment approaches [2, 3, 48, 60, 85]. Predictive modeling is improving the personalization of treatments for insomnia, indicating which patients are likely to respond best to specific therapeutic interventions [63, 86, 87]. Additionally, ML is contributing to our understanding of the relationship between sleep patterns and aging, evaluating sleep quality in chronic disease patients, and predicting cardiovascular risks associated with sleep disorders [56, 88, 89, 90].

Environmental factors affecting sleep, such as ambient noise, are being assessed through ML models to guide interventions that mitigate sleep disturbances. ML‐driven platforms are enhancing sleep education and awareness, promoting proactive management of sleep health [74, 90, 91]. Moreover, ML models are employed to detect sleep onset latency and tailor light therapy for individuals with circadian rhythm disorders, optimizing therapeutic outcomes [57, 74, 84]. Collectively, these advancements highlight ML's broad applicability in enhancing diagnostics, treatment, and understanding of sleep and its disorders, heralding a new era in sleep medicine [45, 58, 66].

## 5 Conclusion

Machine learning (ML) has emerged as a pivotal force in transforming sleep medicine, offering profound innovations from diagnosis and treatment to personalized care and predictive analytics. Through the analysis of vast and complex datasets, ML algorithms have demonstrated exceptional capability in enhancing the accuracy of sleep disorder diagnoses, optimizing treatment efficacies, and unveiling the genetic underpinnings of sleep‐related conditions. The integration of ML with wearable technology has further revolutionized the monitoring and management of sleep disorders, making it more accessible and less invasive for patients. Despite the promise, challenges related to data privacy, ethical considerations, and the need for interdisciplinary collaboration underscore the importance of a cautious, yet optimistic, approach to integrating ML into clinical practice. As we move forward, the continued convergence of ML and sleep medicine holds the potential to significantly improve patient outcomes, highlighting a future where sleep health is increasingly informed by precision medicine and personalized care strategies.

## Author Contributions

**Alexander A Huang:** conceptualization, investigation, funding acquisition, writing – original draft, writing – review and editing, visualization, methodology, validation, software, formal analysis, project administration, resources, supervision, data curation. **Samuel Y Huang:** data curation, supervision, resources, formal analysis, project administration, software, methodology, validation, visualization, writing – review and editing, writing – original draft, funding acquisition, conceptualization, investigation.

## Funding

The authors have nothing to report.

## Conflicts of Interest

The authors declare no conflicts of interest.

## Transparency Statement

The lead author Alexander A. Huang affirms that this manuscript is an honest, accurate, and transparent account of the study being reported; that no important aspects of the study have been omitted; and that any discrepancies from the study as planned (and, if relevant, registered) have been explained.

## Data Availability

Data sharing is not applicable to this article as no datasets were generated or analyzed during the current study. This study is entirely theoretical and does not involve new empirical data.
